# The Clinical and Pathological Effects of Serum C3 Level and Mesangial C3 Intensity in Patients with IgA Nephropathy

**DOI:** 10.1155/2024/8889306

**Published:** 2024-01-02

**Authors:** Xiaoyue Hou, Yanan Liang, Weiwei Zhang, Rong Li

**Affiliations:** Department of Nephrology, The Second Hospital of Tianjin Medical University, Tianjin 300211, China

## Abstract

**Objective:**

To investigate the clinical and pathological effects of serum C3 level, mesangial C3 deposition intensity and blood lipid on IgA nephropathy.

**Methods:**

According to the deposition intensity of immunofluorescence (IF) complement C3 in mesangial region, a total of 151 patients were divided into: (1) negative group (65 cases), (2) weak positive group (51 cases), and (3) strong positive group (35 cases). According to the level of serum C3, the patients were divided into two groups: (1) 33 patients with decreased serum C3 (<85 mg/dL); (2) 118 patients with normal serum C3. The clinicopathological data of the patients were analyzed retrospectively according to the groups.

**Results:**

(1) With the increase of C3 deposition in mesangial region, the mean value of serum C3 level decreased, and the difference was statistically significant (*P*=0.001). (2) Compared with the normal serum C3 group, the blood urea nitrogen (BUN), serum creatinine (Scr), and albumin (Alb) in the serum C3 decreased group were higher, and the differences were statistically significant (*P* < 0.05), while the fasting blood glucose (FBG), low-density lipoprotein (LDL), triglyceride and 24-hr urinary protein (24hUTP) were lower, which difference was statistically significant (*P* < 0.05). (3) Compared with negative group and weak positive group, BUN, uric acid (UA), and Scr were higher in the strong positive group with C3 deposition, while eGFR was lower, with statistical significance (*P* < 0.05). However, C3 deposition in the mesangial region was related to T and enhanced mesangial C3 deposition was associated with more severe tubular atrophy and/or interstitial fibrosis, with statistically significant differences (*P*=0.001).

**Conclusion:**

Patients with strong mesangial C3 deposition and elevated lipid levels had more severe tubule atrophy and/or interstitial fibrosis, as well as more severe pathological lesions, suggesting that activation of the complement system is involved in the pathogenesis of IgA nephropathy and increases the metabolic burden of the kidney.

## 1. Introduction

IgA nephropathy (IgAN) is the most common primary glomerular disease worldwide [1], which is considered as a common cause of end-stage renal failure (ESRF), dialysis, and kidney transplantation. IgAN ranks the first cause of ESRF in China, and is also considered to be the most common cause of end-stage renal disease (ESRD) in young people [2]. IgAN can reduce life expectancy by at least 10 years, and 20%–40% of patients will progress to renal failure [3] 20 years after diagnosis due to the lack of understanding of the pathogenesis of IgAN and effective biomarkers.

The onset of IgAN is often occultic, with various clinical manifestations and varying degrees of severity, which diagnosis is based on renal biopsy pathology. The immunopathological features of IgA1-based immunoglobulin are mainly deposition in the mesangial region and (or) capillary loops of the glomerulus, and may also be accompanied by IgG and (or) IgM deposition, but its pathological manifestations are also complex and varied [4]. With the development of research, some evidence has reported that the replacement pathway of complement and the lectin pathway are also involved in the pathogenesis of IgAN [5, 6]. The activation of complement promotes the occurrence and development of inflammation, and with the deposition of complement C3, long-term local inflammation can lead to chronic glomerular injury and sclerosis, eventually kidney tissue damage.

Previous studies demonstrated that the deposition of complement C3 in the mesangial region seems to be associated with the degree of kidney injury, suggesting that it may have diagnostic value for prognosis [7]. In addition, both complement C3 and C4d have been found to be deposited in the mesangial region, and the deposition of mesangial region C3 exerted better prognostic value than C4d [8]. Compared with asymptomatic IgA deposition, the presence of C3 deposition in renal biopsy is also considered to be strong evidence for IgAN. Although 90% of renal biopsies showed the coexistence of C3 deposition and IgA deposition, some patients did not have C3 deposition in clinical practice. It is worth noting that abnormal lipid metabolism is also related with a meaningfully increased risk of chronic kidney disease progression and development of ESRD [9]. However, the relationship between lipid-related indicators and IgA nephropathy remains unclear. Therefore, whether the density of C3 deposition will affect the clinical and pathological injury remains to be further studied [7]. The purpose of this study was to investigate the effect of serum complement C3 level and mesangial C3 deposition on the clinicopathology of IgA nephropathy patients. We also investigated the relationship between lipid components such as LDL, triglyceride (TG), total cholesterol (TC), high-density lipoprotein (HDL), and IgA nephropathy. In addition, we also discussed the role of complement and complement components in the occurrence and development of IgA nephropathy, providing evidence for the treatment of complement.

## 2. Data and Methods

### 2.1. General Data

A total of 158 patients with primary IgA nephropathy diagnosed by percutaneous renal biopsy in the Department of Nephrology, Second Hospital of Tianjin Medical University from August 2009 to April 2018 were retrospectively collected. Inclusion criteria: (1) There was significant diffuse deposition of IgA in the mesangial region of immunofluorescence (IF), and the histopathology met the diagnostic criteria for IgAN [10]. The clinical manifestations were recurrent gross hematuria or microscopic hematuria, accompanied by proteinuria of different degrees, and some patients could have severe hypertension or renal insufficiency; (2) the number of glomeruli in renal tissue under a light microscope is not less than 10. Exclusion criteria: (1) Combined with secondary glomerular disease: Henoch–Schonlein purpura (HSP) nephritis, hepatitis B related nephritis, lupus nephritis (LN), etc.; (2) excluding diabetic nephropathy (DN), essential hypertension (EP), obesity-related nephropathy, pregnancy-induced toxic kidney injury, etc.; (3) renin-angiotensin system (RAS) blockers, hormones, and immunosuppressants were used before renal biopsy; (4) patients with malignant tumors; (5) incomplete laboratory examination results and pathological data. According to the above criteria, 151 patients were included in this study, including 67 males (44.4%) and 84 females (55.6%).

### 2.2. Methods

Baseline demographic indicators, including sex, age, height, body mass index (BMI), blood pressure (BP), duration of disease, and past medical history, were recorded. The following laboratory data before renal biopsy were collected from 151 patients: fasting plasma glucose (FPG), blood urea nitrogen (BUN), uric acid level (UA), serum creatinine (serum creatinine) Scr, albumin (Alb), TC, low-density lipoprotein cholesterin (LDL-C), HDL, triglyceride (TG), serum immunoglobulin A (IgA), serum immunoglobulin G (IgG), serum immunoglobulin M (IgM), serum complement C3, 24-hr urinary total protein (24hUP), urinary sediment red blood cell count, etc.

According to the serum C3 level grouping standard [11]: patients were divided into a low-C3 group (serum C3 <90 mg/dL) and a normal C3 group (serum C3 ≥ 90 mg/dL). According to the deposition strength of C3 in the mesangial region, patients were divided into three groups [7]: the deposition strength of C3 in the negative group was (− ∼ ±), that of C3 in the weak positive group was (+ ∼ ++), and that of C3 in the strong positive group was (+++ ∼ ++++).

### 2.3. Estimated-Glomerular Filtration Rate (eGFR)

The MDRD (modification of diet in renal disease equation) formula suitable for Chinese population was used for calculation [12], eGFR/(mL (min · 1.73 m^2^)) = 175 × (Scr)^−1.234^ × (age)−^0.179^ × (female × 0.79).

### 2.4. Pathological Oxford Classification

The renal puncture tissues of 151 patients included in the study were examined by light microscopy, electron microscopy and IF. The MEST-C [13] (mesangial-cell score (M), capillary-cell proliferation (E), segmental glomerulosclerosis or adhesion (S), tubule atrophy and interstitial fibrosis (T), crescent body (C)) score was performed according to the Oxford typing standard. The details were as follows: according to the mesangial-cell proliferation (M), the mesangial-cell proliferation >50% was recorded as M1, and the mesangial-cell proliferation >50% was recorded as M0; according to the proliferation of capillary endothelial cells (E), the presence of endothelial-cell proliferation was recorded as E1, and the absence of endothelial-cell proliferation was recorded as E0; according to the presence or absence of segmental glomerular sclerosis (S), the presence of segmental glomerular sclerosis (S) was written as S1 and the absence of segmental glomerular sclerosis (S) was written as S0. Based on the degree of tubule atrophy or interstitial fibrosis (T), T0 was defined as ≤25%, T1 was defined as 26%–50%, and T2 was defined as greater than 50%. The proportion of cellular or fibrotic crescents (C) was divided into three groups: no crescents (C0), <25% crescents (C1), and ≥25% crescents (C2).

### 2.5. Statistic Analysis

All data in this study were statistically analyzed using SPSS17.0 software. The normal distribution and homogeneity of variance were tested for the data of numerical variables. Measurement data with normal distribution were expressed by mean ± standard deviation (*x ± s*), measurement data with nonnormal distribution were expressed by median and interquartile spacing, and counting data were expressed by frequency and percentage. Two independent samples *t* test and one-way ANOVA test were used to compare normal distribution of measurement data. Mann–Whitney *U* test and Wilcoxon rank sum test were used to compare nonnormal distribution of measurement data. Chi-square test or Fisher exact probability method were used to compare the two groups of counting data. When *P* < 0.05 was tested on both sides, the difference was considered statistically significant.

## 3. Results

### 3.1. Serum C3

#### 3.1.1. General Data of Patients in Decreased Serum C3 and Normal C3 Group

The general data of patients in serum C3 decreased group and C3 normal group were shown in Table 1. There were no significant differences in age, gender, course of disease, height, weight, BMI, systolic blood pressure (SBP), and diastolic blood pressure (DBP) between the two groups (*P* ≥ 0.05).

#### 3.1.2. Comparison of Clinical Indexes between Serum C3 Decreased Group and C3 Normal Group

The results showed that the fasting plasma glucose (FPG), BUN, serum creatinine (Scr), and albumin (ALB) levels were higher, and LDL, triglyceride (TG), and 24hUP levels were lower, all of which had statistical differences (*P* < 0.05). However, there was no significant difference in UA, eGFR, TC, HDL, serum IgA, serum IgG, serum IgM, and IgA/C3 between the two groups (*P* ≥ 0.05), as shown in Table 2.

#### 3.1.3. Comparison of Oxford Classification between Serum C3 Reduced Group and Normal Group

**Figure 1:**
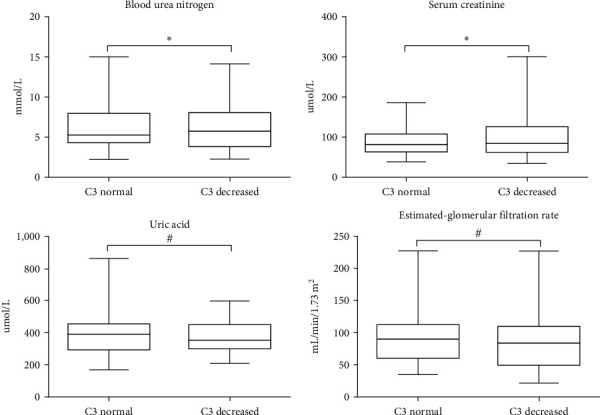
Comparison of serum urea nitrogen, creatinine, uric acid, and eGFR between normal C3 group and low-C3 group. ( ^*∗*^*P* < 0.05,  ^*∗∗*^*P* < 0.01, and ^#^*P* ≥ 0.05, C3 normal group vs. decreased group).

As shown in Table 3, there were no significant differences in the pathological characteristics of the Oxford classification of renal pathological changes between the serum C3 decreased group and the normal C3 group: mesangial proliferation, endothelial-cell proliferation, segmental glomerulosclerosis, tubular atrophy, and/or interstitial fibrosis, and crescent body proportion (*P* ≥ 0.05). As shown as Figure 1.

### 3.2. C3 Deposition in Mesangial Region

The 151 subjects included in this study were divided into 65 cases (43.05%) in the negative group, 51 cases (33.78%) in weak positive group, and 35 cases (23.18%) in strong positive group according to the intensity of C3 deposition in mesangial region.

#### 3.2.1. Mesangial C3 Deposition and General Data of Patients

As shown in Table 4, the negative, weakly positive, and weakly positive groups with C3 deposition in the mesangial region had no correlation with age, gender, course of disease, height, weight, and BMI, but the difference in SBP of patients with different C3 deposition intensity was statistically significant (*F* = 4.919, *P*=0.009). The results showed that the SBP in the negative C3 deposition group was lower than that in the strong positive group (mean difference −8.686, *P*=0.010), and the SBP in the weak positive group was lower than that in the strong positive group (mean difference −10.415, *P*=0.003). Similarly, there was a statistically significant difference in DBP between groups with different C3 deposition strengths (*F* = 5.199, *P*=0.007). Comparison between groups showed that the DBP in the negative C3 deposition group in the mesangial region was lower than that in the strong positive group (mean difference −6.684, *P*=0.004). The DBP in the weak positive group was lower than that in the strong positive group (mean difference was −6.797, *P*=0.005).

#### 3.2.2. C3 Deposition in Mesangial Area and Laboratory Examination Indexes

There were no significant differences in FBG, serum Alb, cholesterol, LDL, HDL, triglyceride, serum IgA, serum IgG, serum IgM, and 24hUP between the three groups (*P* ≥ 0.05). BUN, UA, Scr, eGFR, serum C3, and IgA/C3 ratio were correlated among the three groups, and the differences were statistically significant (*P* < 0.05).

**Figure 2:**
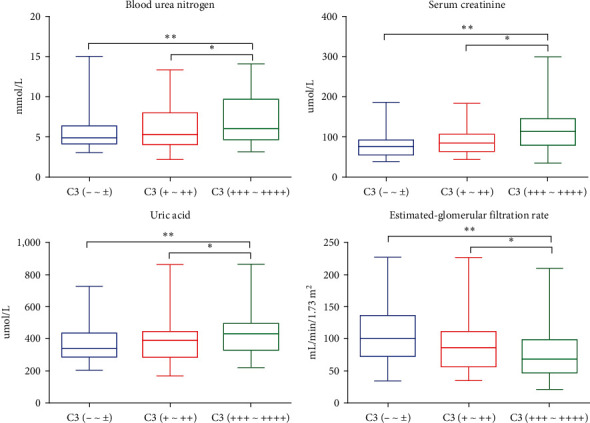
Comparison of blood urea nitrogen, creatinine, uric acid and eGFR in negative, weak positive and strong positive groups of C3 deposition in mesangial region. ( ^*∗*^*P* < 0.05,  ^*∗∗*^*P* < 0.01, negative group vs. strong positive, and weak positive vs. strong positive).

There was statistically significant difference in BUN among the three groups, *F* = 5.98 (*P*=0.003); the results of intergroup comparison showed that the BUN level in the mesangial C3 deposition negative group was lower than that in the strong positive group (mean difference −1.84, *P*=0.001), and the BUN level in the weak positive group was lower than that in the strong positive group (mean difference −1.37, *P*=0.016). The difference of serum uric acid among the three groups was statistically significant, *F* = 4.26 (*P*=0.016). The results of intergroup comparison showed that the blood UA in the negative group with C3 deposition in mesangial region was lower than that in the strong positive group (mean difference −72.02, *P*=0.004), and the blood UA in the weak positive group was lower than that in the strong positive group (mean difference −52.78, *P*=0.044). There was a statistically significant difference in serum creatinine among the three groups, *F* = 9.38 (*P*=0.001). The results of intergroup comparison showed that the serum creatinine level in the mesangial C3 deposition negative group was lower than that in the strong positive group (mean difference −34.20, *P*=0.001), and the serum creatinine level in the weak positive group was lower than that in the strong positive group (mean difference −22.83, *P*=0.006). There was a statistically significant difference in eGFR among the three groups, *F* = 5.95 (*P*=0.003). The results of intergroup comparison showed that the negative group with mesangial C3 deposition had a higher eGFR than the strong positive group (mean difference 8.87, *P*=0.001), and the weak positive group had a higher eGFR than the strong positive group (mean difference 9.29, *P*=0.038). There was a statistically significant difference in serum C3 among the three groups, *F* = 15.50 (*P*=0.001). The results of intergroup comparison showed that the serum C3 level in the negative group with mesangial C3 deposition was higher than that in the weak positive group (mean difference 15.43, *P*=0.002), and the serum C3 level in the negative group with mesangial C3 deposition was higher than that in the strong positive group (mean difference 29.22, *P*=0.002). *P*=0.001), the level of serum C3 in the weak positive group was higher than that in the strong positive group (mean difference was 13.79, *P*=0.015). The difference in serum IgA/C3 ratio between the three groups was statistically significant, *F* = 6.90, (*P*=0.001), and the results of intergroup comparison showed that the IgA/C3 ratio in the negative group with mesangial C3 deposition was lower than that in the strong positive group (mean difference was −1.40, *P*=0.001). The IgA/C3 ratio in the weak positive group with C3 deposition in the mesangial region was lower than that in the strong positive group (mean difference was −0.97, *P*=0.015), and the specific results were shown in Table 5. The statistical quantitative analysis is shown in Figure 2.

#### 3.2.3. Relationship between C3 Deposition in Mesangial Region and Oxford Classification

**Figure 3:**
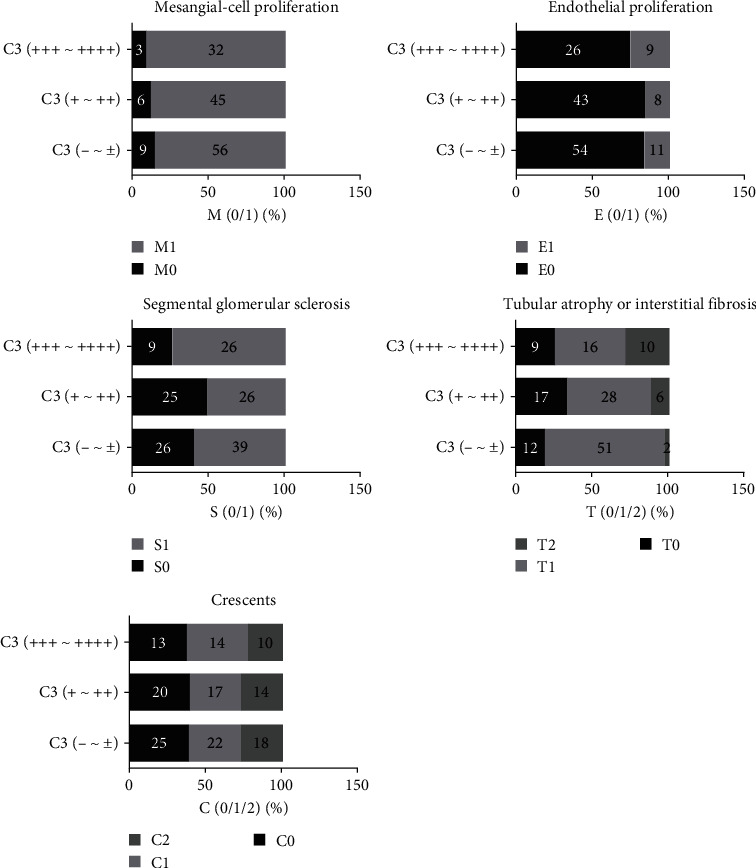
Effect of C3 deposition intensity in mesangial region on mesangial proliferation (M), endothelial-cell proliferation (E), segmental glomerulosclerosis (S), tubular atrophy and/or interstitial fibrosis (T), and crescent body (C).

The results in Table 6 showed that the intensity of C3 deposition in mesangial region had no significant correlation with mesangial proliferation, endothelial-cell proliferation, segmental glomerulosclerosis, and crescent body proportion, but was significantly correlated with the tubular atrophy and/or interstitial fibrosis, with statistically significant differences between groups (*P* < 0.01) as shown in Figure 3.

## 4. Discussion

IgAN is the most widely occurring primary glomerulonephritis in the world. It is the primary cause of end-stage renal disease. Current research progress has shown that the pathogenesis of IgAN is a multistrike pathogenesis model [14]. Deposition of macromolecule IgA1 in mesangial region is the main pathological feature of IgAN [15], and concomitant deposition of C3 is a common manifestation of immunofluorescence staining in renal tissue [2]. Pributin can be detected in the kidney tissue and urine of IgAN patients, and its level is significantly positive; correlated with the urinary protein, Scr, and other levels [5], suggesting that the replacement pathway of complement may be involved in the pathogenesis of IgAN. Similarly, in vitro experimental results confirm that IgA can indeed activate the replacement pathway of complement [6]. In addition to the complement replacement pathway, studies have shown that IgA can bind MBL to activate the lectin pathway [16], suggesting that the activation of the lectin pathway may also be involved in the pathogenesis of IgAN. Based on the above research results, the determination of complement pathway factors and their fragments in kidney tissue, serum, and urine is expected to be a specific marker for the identification of IgAN, and bring ideas for reflecting disease activity and prognosis and specific treatment. It was found in studies that nearly 20% of IgAN patients had hypoc3-emia, and the incidence of ESRD or creatinine doubling was significantly higher than those with normal C3, indicating that the activation of complement has an important impact on the renal prognosis of patients with IgA nephropathy [17]. Nasri et al. [18] found that the activation of complement C3 was related to the increase of urinary protein content and the decrease of renal function in IgAN patients, suggesting that complement activation played a pivotal role in the mechanism of kidney injury in IgAN patients. Nasri et al. [18] followed 528 patients with IgAN for an average of 3 years, and the study results showed that Kaplan–Meier (KM) analysis showed that the median survival time of kidneys in the group with decreased C3 was significantly lower than that in the group with normal C3. What is more, mesangial deposition of C3 and C4d was evaluated concurrently. C3 showed better predictability than C4d, suggesting that the lectin pathway alone has limited clinical prognostic value [8]. Patients with native renal biopsies with histologically diagnosed cases of proliferative glomerular diseases exposed that the caseload of C4d positivity was higher than C3 positivity patents [19]. The results of retrospective analysis in this study showed that 21.85% of patients with IgA nephropathy had hypoc3-emia, and the mean value of BUN and serum creatinine in patients with reduced serum C3 were higher than those in the normal group, with significant statistical significance, suggesting that C3 consumption in IgAN patients was closely related to renal function damage. The decrease of serum C3 can reflect the impairment of renal function to some extent and may represent a poor prognosis, which was consistent with the previous findings.

Deposition of C3 at different intensities in the mesangial region is a common histological feature in IgA nephropathy. Coppo and D'Amico [20] found that with the enhancement of C3 deposition in the mesangial region, the kidney lesions in IgAN patients showed an aggravating trend and were associated with the worse renal prognosis. Some clinical patients have IgA deposition without C3 deposition. In this study, compared with patients with strong negative C3 deposition in mesangial region, BUN, UA, Scr, and eGFR values of patients with strong positive C3 deposition in the mesangial region were higher than those of negative and weak positive C3 deposition in the mesangial region with a statistically significant difference, suggesting that with the enhancement of C3 deposition in mesangial region, the damage of renal function was more serious, which indicated that C3 was an important indicator. This phenomenon can be explained by the theory that the terminal pathway of complement activation is completed in kidney tissue. Currently, experiments have detected C5, C6, C9, and MAC in kidney biopsy tissue, providing further evidence for the explanation and rationality of this phenomenon. IgAN is characterized by the deposition of IgA and complement C3, often with IgG and/or IgM, in the glomerular mesangial region, followed by mesangial-cell proliferation and extreme synthesis of extracellular matrix in glomeruli [21]. What is more, IgAN is described by mesangial deposits of IgA1-containing immune complexes. IgA1 frequently codeposits with complement C3 and adjustable IgG and/or IgM [22].

The results of this study showed that with the increase of C3 deposition intensity in the mesangial region, the serum C3 level decreased, and the difference was statistically significant, consistent with the research conclusion of Zhu et al. [23], which verified that the activation and consumption of complement C3 existed in the pathogenesis of IgAN, and the activation of complement C3 might be involved in aggravating the disease progression of IgAN. Moreover, the decrease of serum C3 level can also reflect the degree of kidney damage in IgAN patients to a certain extent. At present, some studies have reported that the serum IgA/C3 ratio can be used as an important indicator to evaluate the prognosis and disease activity of IgAN [24], and specifically pointed out that serum IgA/C3 ≥ 3.2 was an effective predictor of ESRD in IgAN patients in Chinese population [24]. In this study, the IgA/C3 ratio of the strong positive group with mesangial C3 deposition was significantly higher than that of the negative group and the weak positive group, indicating the occurrence of poor prognosis. Moreover, enhanced mesangial C3 deposition in this study was associated with the increased tubular atrophy and/or interstitial fibrosis, suggesting more severe pathological manifestations and poorer renal prognosis.

In addition, there are still some limitations and shortcomings in this study. First, the sample size of this study is limited, which may lead to a segmentary conclusion. What is more, a large sample verification is necessary via multicenter and large-sample studies. Third, the effect of serum C3 levels and the intensity of C3 deposition in the mesangial region on long-term prognosis were not clarified in this cross-sectional study. In the follow-up work, we will follow-up on the serum C3 levels of the patients to further confirm whether serum C3 reduction and mesangial C3 deposition have the potential to predict prognosis in patients with IgAN. Furthermore, we will take further steps to expand the sample size and conduct multiple heart studies to confirm our findings in multiple study cohorts.

## 5. Conclusion

In this study, it was found that patients with decreased serum C3 and strong C3 deposition in the mesangial region had more severe clinical indicators of renal function. What is more, such patients were usually accompanied by more serious pathological damage such as renal tubular atrophy and (or) interstitial fibrosis, demonstrating that serum C3 could be applied as a potential biological marker for the occurrence and prognosis of IgAN disease, which provides a new strategy and direction for early clinical monitoring. Early targeted inhibition of serum C3 transformation may reduce deposition and subsequent pathological damage. In addition, the decrease of serum C3 and the deposition of the mesangial region were associated with the activation of the complement system, suggesting that the activation of the complement system was involved in the pathogenesis and exacerbation of IgA nephropathy.

## Figures and Tables

**Table 1:** General data of C3 reduced group and C3 normal group.

Characteristics	C3 decreased group (<90 mg/dL)	C3 normal group (≥90 mg/dL)	*P* Value
Age (year)	38.47 ± 12.28	34.88 ± 8.74	0.058
Female, *n* (%)	23 (27.4%)	61 (72.6%)	0.076
Duration from onset (d)	180 (515)	347 (1,065)	0.395
Height (cm)	168.44 ± 8.11	167.3 ± 9.78	0.344
Weight (kg)	71.9 ± 14.4	62.27 ± 11.76	0.105
BMI (kg/m^2^)	25.22 ± 4.08	22.16 ± 3.09	0.057
SBP (mmHg)	128.96 ± 16.07	132.03 ± 17.16	0.660
DBP (mmHg)	84.29 ± 11.01	86.36 ± 11.56	0.929

Abbreviations. BMI, body mass index; SBP, systolic blood pressure; DBP, systolic blood pressure.

**Table 2:** Clinical indicators of patients with decreased C3 and normal C3 group.

Laboratory examination	Serum C3 decreased group (<90 mg/dL)	Normal C3 group (≥90 mg/dL)	*P* Value
FPG (mmol/L)	5.01 ± 0.4	5.2 ± 0.97	0.002
BUN (mmol/L)	6.6 ± 3.39	6.02 ± 2.4	0.006
UA (umol/L)	378.73 ± 112.51	389.09 ± 123.4	0.816
Scr (umol/L)	98.78 ± 52.3	89.67 ± 35.44	0.035
eGFR (mL/min/1.73 m^2^)	93.06 ± 51.6	95.62 ± 41.43	0.258
Alb (g/L)	38.2 ± 6.3	36.33 ± 9.17	0.009
TC (mmol/L)	5.72 ± 1.76	6.29 ± 2.5	0.085
LDL-C (mmol/L)	3.63 ± 1.39	3.99 ± 2.09	0.046
HDL (mmol/L)	1.32 ± 0.42	1.24 ± 0.42	0.920
TG (mmol/L)	1.58 ± 0.7	2.4 ± 1.66	0.011
Serum IgA (mg/L)	329.36 ± 93.84	342.19 ± 153.67	0.108
Serum IgG (mg/L)	911.15 ± 392.61	1012.2 ± 386.87	0.492
Serum IgM (mg/L)	118.45 ± 55.91	110.05 ± 50.83	0.663
24hUP (g/24 hr)	2.34 ± 1.41	3.89 ± 3.17	0.001
IgA/C3 ratio	4.65 ± 2.21	3.03 ± 1.6	0.702

Abbreviations. FPG, fasting plasma glucose; BUN, blood urea nitrogen; UA, uric acid level; Scr, serum creatinine; Alb, albumin; TC, total cholesterol; LDL-C, low-density lipoprotein cholesterin; HDL, high-density lipoprotein cholesterin; TG, triglyceride; 24hUP, 24-hr urinary total protein, 24hUP.

**Table 3:** Pathological characteristics of renal biopsy in patients with decreased C3 and normal C3.

Stage	Serum C3 < 90 mg/dL	Serum C3 ≥ 90 mg/dL	*P* Value
M0	1	17	0.060
M1	32	101	
E0	25	98	0.325
E1	8	20	
S0	13	47	0.565
S1	20	71	
T0	11	27	0.791
T1	14	81	
T2	8	10	
C0	11	47	0.779
C1	14	39	
C2	8	32	

**Table 4:** C3 deposition and general data.

Characteristics	Negative (65)	Weakly positive (51)	Strongly positive (35)	*P* Value
Age (year)	39.78 ± 13.52	35.65 ± 10.42	36.77 ± 9.04	0.144
Female, *n* (%)	38/65 (58.46%)	29/51 (56.86%)	17/35 (48.57%)	0.392
Male, *n* (%)	27/65 (41.54%)	22/51 (43.14%)	18/35 (51.43%)	0.392
Duration from onset (d)	365 (1060)	180 (1065)	180 (670)	0.243
Height (cm)	167.46 ± 8.35	167.67 ± 8.32	170.31 ± 8.84	0.239
Weight (kg)	70.61 ± 14.73	68.64 ± 14.62	69.98 ± 13.71	0.765
BMI (kg/m^2^)	25.11 ± 4.4	24.22 ± 3.88	23.99 ± 3.67	0.337
SBP (mmHg)	128.2 ± 15.6	126.47 ± 15.12	136.89 ± 17.46	0.009
DBP (mmHg)	83.23 ± 10.55	83.12 ± 10.01	89.91 ± 12.4	0.007

*Note*: In the above data, the disease course was marked in the form of median (interquartile distance), and the remaining data were represented by *x* ± s. Abbreviations: BMI, body mass index; SBP, systolic blood pressure; DBP, systolic blood pressure.

**Table 5:** C3 deposition in mesangial area and laboratory examination indexes.

Laboratory examination	Negative	Weakly positive	Strongly positive	*P* Value
FPG (mmol/L)	5.32 ± 1.02	5.03 ± 0.84	5.16 ± 0.55	0.107
BUN (mmol/L)	5.56 ± 2.15	6.04 ± 2.69	7.4 ± 3.04	0.003
UA (umol/L)	363.63 ± 104.07	382.87 ± 129.73	435.65 ± 125.5	0.016
Scr (umol/L)	79.89 ± 30.7	91.27 ± 37.21	114.1 ± 48.63	0.001
eGFR (mL/min/1.73 m^2^)	105.91 ± 43.39	94.79 ± 43.14	75.32 ± 38.85	0.003
Alb (g/L)	36.14 ± 9.83	36.56 ± 8.04	38.1 ± 7.04	0.552
TC (mmol/L)	6.27 ± 2.61	6.11 ± 2.48	6.06 ± 1.69	0.900
LDL-C (mmol/L)	4.01 ± 2.31	3.85 ± 1.86	3.83 ± 1.32	0.873
HDL (mmol/L)	1.2 ± 0.35	1.32 ± 0.48	1.27 ± 0.44	0.280
TG (mmol/L)	2.31 ± 1.33	1.87 ± 1.01	2.57 ± 2.31	0.098
Serum IgA (mg/L)	336.32 ± 167.42	334 ± 126.01	352.94 ± 115.4	0.813
Serum IgG (mg/L)	1055.91 ± 409.46	933.16 ± 337.26	950.94 ± 412.63	0.192
Serum IgM (mg/L)	114.93 ± 51.41	113.1 ± 58.61	104.45 ± 42.23	0.619
Serum C3 (mg/dL)	120.66 ± 24.9	105.23 ± 25.33	91.43 ± 27.32	0.001
24hUP (g/24 hr)	3.73 ± 3.32	3.65 ± 2.83	3.08 ± 2.34	0.558
IgA/C3 ratio	2.91 ± 1.67	3.34 ± 1.41	4.31 ± 2.42	0.001

Abbreviations. FPG, fasting plasma glucose; BUN, blood urea nitrogen; UA, uric acid level; Scr, serum creatinine; Alb, albumin; TC, total cholesterol; LDL-C, low-density lipoprotein cholesterin; HDL, high-density lipoprotein cholesterin; TG, triglyceride; 24hUP, 24-hr urinary total protein, 24hUP.

**Table 6:** Intensity and pathological characteristics of C3 deposition in mesangial region.

Pathological index	C3 negative group (*n*)	C3 weak positive group (*n*)	C3 strong positive group (*n*)	*P* Value
M0	9	6	3	0.739
M1	56	45	32	
E0	54	43	26	0.454
E1	11	8	9	
S0	26	25	9	0.095
S1	39	26	26	
T0	12	17	9	0.001
T1	51	28	16	
T2	2	6	10	
C0	25	20	13	0.967
C1	22	17	14	
C2	18	14	8	

## Data Availability

All data generated or analyzed during this study are included in this published article.
